# Association between PPARGC1A single nucleotide polymorphisms and increased risk of nonalcoholic fatty liver disease among Iranian patients with type 2 diabetes mellitus

**DOI:** 10.3906/sag-1808-138

**Published:** 2019-08-08

**Authors:** Leila SAREMI, Shirin LOTFIPANAH, Masumeh MOHAMMADI, Hassan HOSSEINZADEH, Zahra HOSSEINI-KHAH, Behrooz JOHARI, Zohreh SALTANATPOUR

**Affiliations:** 1 Department of Biology, Science and Research Branch, Islamic Azad University, Tehran Iran; 2 Farhangian University, Shahid Mofatteh Teacher Education Paradise, Tehran Iran; 3 Department of Biology, Faculty of Science, Yazd University, Yazd Iran; 4 Department of Molecular Medicine, School of Advanced Technologies in Medicine, Tehran University of Medical Sciences, Tehran Iran; 5 Molecular and Cell Biology Research Center, Mazandaran University of Medical Sciences, Sari, Mazandaran Iran; 6 Department of Medical Biotechnology, School of Medicine, Zanjan University of Medical Sciences, Zanjan Iran; 7 Medical Genetics Center, Endocrinology and Metabolism Research Institute, Tehran University of Medical Sciences, Tehran Iran

**Keywords:** Nonalcoholic fatty liver disease, PPARGC1A gene, Gly482Ser polymorphism, type 2 diabetes

## Abstract

**Background/aim:**

Environmental and genetic factors may play a major role in the development of nonalcoholic fatty liver disease (NAFLD) among people with obesity and type 2 diabetes mellitus. Based on the fact that PGC-1α, as the protein encoded by the PPARGC1A gene, plays a key role in energy metabolism pathways, it has been hypothesized that polymorphisms within the PPARGC1A gene may be associated with increased risks of NAFLD. Thus, this study was designed to evaluate the Gly482Ser polymorphism (rs8192678) within the PPARGC1A gene and its association with the increased risk of NAFLD in Iranian patients with type 2 diabetes.

**Materials and methods:**

A total of 145 NAFLD patients with a history of type 2 diabetes and 145 healthy control subjects were included in the study. Gly482Ser polymorphism genotyping was done using the amplification refractory mutation system-polymerase chain reaction (ARMS-PCR) technique.

**Results:**

The results showed a significant difference between the PPARGC1A Gly482Ser polymorphism in NAFLD patients and the healthy controls. Accordingly, the AA genotype and A allele were increased in the NAFLD patients when compared to the healthy controls. However, no significant correlation was observed between the Gly482Ser polymorphism and the physiological and biochemical parameters.

**Conclusion:**

Based on the results, the AA genotype, which is associated with the insertion of Ser, can be considered as a risk factor for the development of NAFLD in Iranian patients with diabetes type 2.

## 1. Introduction

Nonalcoholic fatty liver disease (NAFLD) is one of the most common forms of chronic liver disorder in developed countries, and it affects more than 70% of patients with type 2 diabetes mellitus and people suffering from obesity [1,2]. NAFLD is characterized by hepatic and systemic insulin resistance, dyslipidemia, and persistent abnormalities in the liver enzymes [3–5]. NAFLD comprises a broad spectrum of disease severity, ranging from benign hepatocellular steatosis to nonalcoholic steatohepatitis (NASH-fatty changes with inflammation and hepatocellular damages), advanced liver cirrhosis, hepatocellular carcinoma, and increased risk of liver-related mortalities [6]. 

Inherited factors, along with environmental factors, play an important role in the development of NAFLD [2]. *PPARGC1A* is one of the strongest biological candidate genes for susceptibility to NAFLD. The peroxisome proliferator-activated receptor-gamma coactivator-1 alpha (PGC-1α) protein is encoded by this gene and plays a role in energy metabolism, including insulin resistance, oxidative phosphorylation, hepatic gluconeogenesis, and mitochondrial biogenesis and respiration, which are important in the development of NAFLD [6]. Moreover, there are some reports regarding the role of PGC-1α in the homeostatic control of systemic energy metabolism, regulation of lipid and carbohydrate metabolism, development of obesity, and control of blood pressure [7,8]. 

Recent investigations have indicated that single nucleotide polymorphisms (SNPs) of the *PPARGC1A *gene may be associated with the susceptibility and progression of NAFLD, obesity, type 2 diabetes mellitus, and hypertension in various ethnicities [9,10]. Previous studies reported an association between *PPARGC1A* polymorphism at position +1564G/A (rs8192678) with NAFLD, which is associated with the replacement of Gly with Ser (Gly482Ser) [10–12]. However, this polymorphism in the Iranian population has not yet been studied and needs to be explored. 

Thus, this study was designed to investigate the association between Gly482Ser polymorphism within the *PPARGC1A* gene and increased susceptibility to NAFLD in Iranian subjects.

## 2. Materials and methods

A total of 145 Iranian NAFLD patients and 145 healthy control subjects were recruited to participate in this study. NAFLD was confirmed by liver biopsy in all 145 patients. Exclusion criteria for the NAFLD patients were the presence of either ballooning cells or perisinusoidal/pericellular fibrosis in zone 3 of the hepatic acinus, and lobular inflammation, in addition to steatosis [10]. Moreover, the selection criteria included no history of infections, autoimmunity, alcohol- or drug-induced hepatitis, sclerosing cholangitis, α1-antitrypsin deficiency, primary biliary cirrhosis, hemochromatosis, Wilson’s diseases, or alcohol consumption.

The age- and sex-matched control subjects, who had normal abdominal ultrasonography, were confirmed to have normal liver functions and no metabolic diseases, viral hepatitis, or alcohol abuse. All of the control subjects had normal systolic (<132 mmHg) and diastolic blood pressures (<85 mmHg), total cholesterol levels (<200 mg/dL), fasting glucose (<90–100 mg/dL), and body mass indices (BMIs) (<25 kg/m2). The characteristics of the NAFLD patients and control subjects are presented in Table 1.

**Table 1:** Characteristics of the NAFLD patients and healthy control subjects.

Characteristics	Control	NAFLD	P-value
Men/women	71/74	72/73	0.831
Age (years)	51.3 ± 10	53.9 ± 9	0.423
Weight (kg)	86.3 ± 4.4	89.2 ± 3.8	0.577
Systolic blood pressure (cmHg)	13.12 ± 1.98	13.95 ± 2.48	0.001
Diastolic blood pressure (cmHg)	8.36 ± 0.94	8.02 ±1.18	0.002
BMI (kg/m2)	24.6 ± 2.6	29.4 ± 4.5	0.179
FBS (mg/dL)	89.6 ± 13.1	182.1 ± 20.3	0.572
Total cholesterol (mg/dL)	198.8 ± 34.1	204.1 ± 28.4	0.1
2h plasma glucose (mg/dL)	120.41 ± 12.40	240.19 ± 20.15	0.012
HDL-C (mg/dL)	45.0 ± 7.3	49.0 ± 9.7	0.490
LDL-C (mg/dL)	98.03 ± 12.5	103.0 ± 21.2	0.471
TG (mg/dL)	99 ± 40.4	112 ± 36.09	0.112
Cr (mg/dL)	0.96 ± 0.2	1.02 ± 0.42	0.031
HbA1c (%)	6.23 ± 3.0	8.63 ± 1.2	0.267
Microalbumin (mg/dL)	11.12 ± 5.6	19.03 ± 6.6	0.420

Data are mean ± SD; NAFLD: nonalcoholic fatty liver disease, BMI: body mass index, FBS: fasting blood sugar, HDL-C: high-density lipoprotein-cholesterol, LDL-C: low-density lipoprotein-cholesterol, TG: triglycerides, Cr: creatine.

Written informed consent was obtained from all participants. The study was approved by the institutional ethics committee and conducted in accordance with the Declaration of Helsinki. The body weights of the participants were measured using a calibrated electronic scale, and venous blood samples were obtained after an overnight fast (12 h). The total serum cholesterol, high-density lipoproteins (HDL), hemoglobin A1c (HbA1c), and triglyceride levels were measured via the enzymatic method (commercial Analco kit, Poland). The low-density lipoprotein (LDL) cholesterol levels were calculated using the formula of Friedewald [13].

Genomic DNA was isolated from whole blood samples using the Bioneer DNA extraction kit (AccuPrep Genomic DNA Extraction Bioneer Kit, Republic of Korea). Gly482Ser polymorphism genotyping was performed using the amplification refractory mutation system (ARMS)-polymerase chain reaction (PCR) method. The ARMS technique uses allele-specific sequencing primers and the amplification arrest caused by noncomplementary nucleotide(s), located at the 3’ end of the primer. In this study, a mutation-specific primer was used, resulting in the formation of an additional product only in the presence of the A allele. A total of 3 primers were used, as follows: two forward primers, 5’ GACGAAGCAGACAAGACCG 3’ and 5’ GACGAAGCAGACAAGACCA 3’, and one common reverse primer, 5’ AGAGTTAAAAGAAGAACAAGAAGGAG 3’.

Thermal cycling was carried out under the following conditions: 95 °C for 5 min, and then 30 cycles at 95 °C for 30 s, 56.5 °C for 30 s, and 72 °C for 30 s. This was followed by incubation at 72 °C for 5 min. Agarose gel electrophoresis was used to detect the stained 210-bp PCR products (Figure). 

**Figure:**
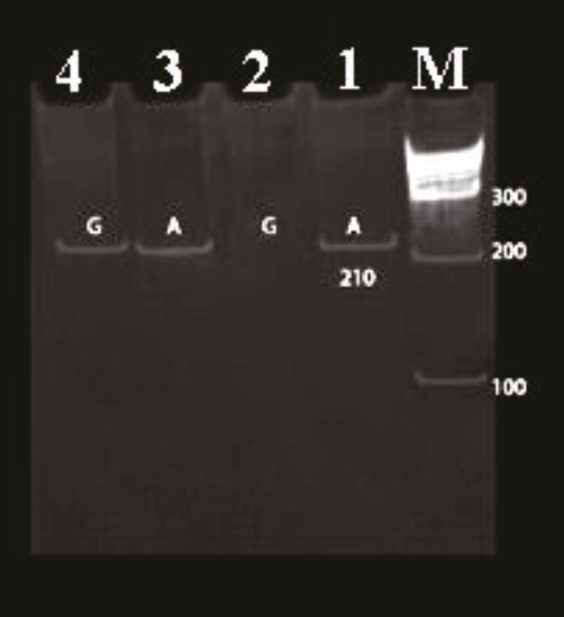
Gly482Ser polymorphism in the PPARGC1A gene. M: 100-bp ladder molecular size marker. Lanes 1 and 4: 210-bp PCR product. Lanes 1 and 2: AA genotype. Lanes 3 and 4: AG genotype.

SPSS software (IBM Corp., Armonk, NY, USA) was used for data analysis. Categorical variables including the frequencies of the genotypes and alleles were compared using chi-square analysis and continuous variables by the Student t-test. The odds ratio (OR) and its 95% confidence interval (CI) were calculated to estimate the strength of the association between patients and controls and the polymorphism genotype alleles. Physical and laboratory data are presented as means ± standard deviations (SDs). P < 0.05 was regarded as statistically significant.

## 3. Results

The genotype and allele frequencies of the Gly482Ser polymorphism are shown in Table 2. Genotypes AA and AG were detected, but no homozygote for the* PPARGC1A* mutation (GG) was found. The frequency of the AA and AG genotypes in the NAFLD group was 120 and 25, respectively, while in the control group, it was 105 and 40, respectively. Data analysis revealed that the genotypes and alleles in both NAFLD patients and control subjects were in Hardy–Weinberg equilibrium (Table 3). The chi-square test showed a significant difference between the allele frequencies of the Gly482Ser polymorphism among the patients and the controls (P < 0.05). As shown in Table 2, the frequency of A alleles in the NAFLD patients and controls was 265 and 150, respectively, while for the G allele it was 25 and 40. The statistical analysis revealed a significant difference between the groups regarding the *PPARGC1A* A and G alleles (P = 0.002).

**Table 2:** Genotypic association of the Gly482Ser polymorphism with NAFLD.

Variable	Genotype n = 145		OR (95% CI)	P-value
	AA	AG		
NAFLD patients	120	25	1.829 (1.04–3.215)	0.035
Control subjects	105	40		
Variable	Alleles		OR (95% CI)	P-value
	A	G		
NAFLD patients	265	25	2.01 (1.41–3.47)	0.002
Control subjects	250				40

**Table 3:** Observed and expected frequencies among the NAFLD patients and control subjects using Hardy–Weinberg equilibrium.

Groups	Genotypes		Allele A	Allele G	Observed frequency	Expected frequency	P-value
NAFLD patients	AA	120	240	0	120	121.08	0.2560
	AG	25	25	25	25	22.84	
	GG	0	0	0	0	1.08	
	Total	145	265	25	145	145.00	
Controls	AA	105	210	0	105	107.76	0.0540
	AG	40	40	40	40	34.48	
	GG	0	0	0	0	2.76	
	Total	145	250	40	145	145.00	

As shown above, the frequencies of either the genotypes or alleles in both groups were in Hardy–Weinberg equilibrium.

The significant differences in the genotype and allele distributions between the two groups (P < 0.01) suggested that there was a significant relationship between the distribution of this polymorphism and NAFLD status. The heterozygote AA genotype was associated with a significantly higher risk of NAFLD (P < 0.01; adjusted OR = 1.829, 95% CI = 1.04–3.215) compared with the AG genotype. Statistical analysis also revealed that the A allele in NAFLD patients was significantly associated with increased risks of NAFLD (adjusted OR = 2.01, 95% CI = 1.41–3.47) in our population (P**= 0.002). 

Analysis of the association between the two genotypes with the physiological and biochemical parameters in the patients and controls is presented in Table 4, where it can be seen that no significance difference was found in these parameters (BMI, FBS, HbA1c, macroalbuminuria, creatine, and lipid profile [TG, TC, HDL-C, and LDL-C] levels) and the AA and AG genotypes between the patients and controls. However, total cholesterol, LDL, and HDL levels can be considered as independent risk factors for NAFLD.

**Table 4:** Analysis of the physiologic variables in the control and case groups.

Variable	Control subjects			NAFLD patients			AA	AG	P-value	AA	AG	P-value
	N	105	40	-	120	25	-
BMI (kg/m2)	24.6 ± 3.5	24.5 ± 3.4	0.86	29.4 ± 4.7	29.2 ± 4.4	0.75
FBS (mg/dL)	89.6 ± 9.1	87.4 ± 9	0.15	190.2 ± 20.2	182.1 ± 20.3	0.57
Total cholesterol (mg/dL)	48 ± 1.6	44.7 ± 1.7	0.2	42.2 ± 1.8	48.5 ± 1.8	0.92
HDL-C (mg/dL)	39.7 ± 10.3	39.7 ± 11.8	0.96	52.4 ± 11.9	49.7 ± 11.9	0.2
LDL-C (mg/dL)	50.6 ± 13.3	52.4 ± 12	0.4	88.1 ± 32.2	89.9 ± 35.3	0.76
TG (mg/dL)	76.5 ± 1.5	71.9 ± 1.5	0.99	110.1 ± 2	104.2 ± 2	0.77
Cr (mg/dL)	1 ± 0.3	1.1 ± 0.4	0.52	1 ± 0.3	1 ± 0.31	0.61
HbA1c (mg/dL)	6.8 ± 1.5	6.4 ± 1.6	0.76	8.3 ± 1.9	8.5 ± 2	0.90
Microalbumin (mg/dL)	6.3 ± 1.9	6.8 ± 2.1	0.18	20.7 ± 7	20.6 ± 6.8	0.91

Data are shown as mean ± SD; NAFLD: nonalcoholic fatty liver disease, BMI: body mass index, FBS: fasting blood sugar, HDL-C: high-density lipoprotein-cholesterol; LDL-C: low-density lipoprotein-cholesterol, TG: triglycerides, Cr: creatine.

## 4. Discussion

NAFLD is a major public health problem and a hepatic manifestation of the metabolic syndrome, with an even higher risk in individuals with obesity and type 2 diabetes [1,2]. Patients with NAFLD and type 2 diabetes present higher levels of complications of diseases, including liver cirrhosis, hepatocellular carcinoma, and death [14].

The protein encoded by the *PPARGC1A* gene (PGC-1α) is a transcriptional factor that moderates the genes involved in lipid and energy metabolism [10,11]. Previous studies indicated that a deficiency of this protein results in enhanced lipogenesis and hepatic steatosis. PGC-1α interacts with PPAR-α, which is highly expressed in the liver and coordinates the induction of fatty acid oxidation in response to fasting. It also binds to PPAR-γ and activates it to induce the expression of genes that are involved in the differentiation of brown fat tissues. The binding of PGC-1α to PPAR-γ permits the interaction of this protein with multiple transcription factors [15]. PGC-1α also plays a major regulatory role in mitochondria biogenesis and glucose/fatty acid metabolism. Recent studies have suggested the downregulation of PGC-1α-responsive genes in insulin-resistant and type 2 diabetic subjects [16]. Patti et al. analyzed the gene expression of PGC-1α in diabetic Mexican-American subjects. They found that insulin resistance and type 2 diabetes were associated with decreased levels of PGC-1α in the skeletal muscle in both diabetic and family history-positive nondiabetic subjects [6]. Thus, genetic factors that alter the expression of the molecule may participate in the pathogenesis of NAFLD.

In recent years, numerous related case-control gene- and allele-associated studies have investigated the association between single nucleotide polymorphisms and the risk of disease in NAFLD patients. Particularly, a common Gly482Ser polymorphism (rs8192678) of the *PPARGC1A* gene was reported to be related to the susceptibility of NAFLD in some ethnicities [10]. This polymorphism may reduce PGC-1α activities and/or may change its interaction with other transcription factors required to regulate oxidative stress and lipid metabolism, which finally triggers the pathogenesis of NAFLD in type 2 diabetic subjects. Gly482Ser polymorphism has also been widely reported to be associated with an increased risk of insulin resistance, obesity, and type 2 diabetes in some populations [17–19]. 

Interestingly, the results of the current investigations confirmed the plausible role of the Gly482Ser polymorphism in the Iranian population. Accordingly, the results revealed that both the AA genotype and A alleles were significantly associated with a higher risk of NAFLD. Additionally, based on the statistical analysis, the AA genotype and A allele increased the risk of NAFLD by 1.82- and 2-fold when compared to the AG genotype and G allele, respectively, in Iranian patients with type 2 diabetes. In parallel with our results, several association studies have demonstrated that the Gly482Ser polymorphism increases the susceptibility to NAFLD in type 2 diabetic patients [17–19]. This genotype distribution suggests a significant association between *PPARGC1A* mutations and NAFLD status. For example, Muller et al. reported that the AA genotype could be associated with early insulin secretion and altered lipid oxidation in a Pima Indian population [18]. SNPs of the *PPARGC1A* gene were also shown to be related to NAFLD in obese Taiwanese children [3]. Saremi et al. observed that the rs8192678 SNP of the *PPARGC1A* gene contributed to the onset of type 2 diabetes in Danish patients [4]. However, a study by Hui et al. did not find any association between the Gly482Ser variants and NAFLD in Han Chinese people [20]. 

Collectively, based on our results, it seems that the AA genotype and A allele can be considered important risk factors for the induction of NAFLD in the Iranian population. 

A stratified analysis to assess the coimpacts of Gly482Ser variations with other known risk factors for NAFLD was accomplished here. For this purpose, the associations of the two genotypes with the physiological and biochemical variables, including BMI, FBS, creatine, TG, plasma lipid levels, HbA1c, and microalbumin levels, were investigated. The results revealed that none of the biochemical parameters were associated with the Gly482Ser polymorphism. Thus, it may be concluded that although Gly482Ser variations were associated with an increased risk of NAFLD in the Iranian population, their role regarding the metabolism of lipids and glucose needs to be explored by further investigation.

The limitation of this study was the low number of diabetic patients with NAFLD available for the analysis. Large-scale well-designed studies, matched case-controls, and functional studies are great tools to support these findings.

In conclusion, the results showed that the *PPARGC1A* Gly482Ser polymorphism has an impact on NAFLD susceptibility in the Iranian population. As a result, it can be concluded that this gene polymorphism has an important genetic contribution to the etiology of NAFLD in patients with diabetes.

## Acknowledgment

This study was supported by the Medical Genetics Center, Endocrinology and Metabolism Research Institute, Tehran University of Medical Sciences, Tehran, Iran.

## References

[ref0] (2015). Nonalcoholic steatohepatitis: emerging targeted therapies to optimize treatment options. Drug Design, Development and Therapy.

[ref1] (2016). Effects of obesity on hormonally driven cancer in women. Science Translational Medicine.

[ref2] (2006). Relations between carotid artery wall thickness and liver histology in subjects with nonalcoholic fatty liver disease. Diabetes Care.

[ref3] (2016). Association of HFE gene mutations with nonalcoholic fatty liver disease in the Iranian population. Cellular and Molecular Biology (Noisy-le-.

[ref4] (2019). The Pro12Ala polymorphism in the PPAR-γ2 gene is not associated with an increased risk of NAFLD in Iranian patients with type 2 diabetes mellitus.

[ref5] (2010). Lack of association between peroxisome proliferator-activated receptors alpha and gamma2 polymorphisms and progressive liver damage in patients with non-alcoholic fatty liver disease: a case control study. BMC Gastroenterology.

[ref6] (2016). Treatment of nonalcoholic fatty liver disease (NAFLD) in patients with type 2 diabetes mellitus. Clinical Diabetes and Endocrinology.

[ref7] (2002). Expanding the natural history of nonalcoholic steatohepatitis: from cryptogenic cirrhosis to hepatocellular carcinoma. Gastroenterology.

[ref8] (2005). The natural history of nonalcoholic fatty liver disease: a population-based cohort study. Gastroenterology.

[ref9] (2008). Association between PPARGC1A polymorphisms and the occurrence of nonalcoholic fatty liver disease (NAFLD). BMC Gastroenterolgy.

[ref10] (2013). Maternal high-fat intake during pregnancy programs metabolic-syndrome-related phenotypes through liver mitochondrial DNA copy number and transcriptional activity of liver PPARGC1A. Journal of Nutritional Biochemistry.

[ref11] (2015). Relation between PPARGC1A gene polymorphisms with the increased risk of coronary artery disease among patients with type 2 diabetes mellitus in Iran. General Endocrinology.

[ref12] (1972). Estimation of the concentration of low-density lipoprotein cholesterol in plasma, without use of the preparative ultracentrifuge. Clinical Chemistry.

[ref13] (2017). Non-alcoholic fatty liver disease: a clinical update. Journal of Clinical and Translational Hepatology.

[ref14] (2006). PGC-1α: a key regulator of energy metabolism. Advances in Physiology Education.

[ref15] (2004). Impaired mitochondrial activity in the insulin-resistant offspring of patients with type 2 diabetes. New England Journal of Medicine.

[ref16] (2002). A genetic variation in the PGC-1 gene could confer insulin resistance and susceptibility to type II diabetes. Diabetologia.

[ref17] (2003). A Gly482Ser missense mutation in the peroxisome proliferator-activated receptor gamma coactivator-1 is associated with altered lipid oxidation and early insulin secretion in Pima Indians. Diabetes.

[ref18] (2009). Differential proteomic analysis of STAT6 knockout mice reveals new regulatory function in liver lipid homeostasis. Journal of Proteome Research.

